# Telemedicine in Times of Crisis—A SWOT Assessment Based on Romanian Healthcare Professionals’ Perceptions

**DOI:** 10.3390/healthcare13192474

**Published:** 2025-09-29

**Authors:** Gianina-Valentina Băcescu Ene, Corina Mărginean, Damiana-Maria Vulturar, Corina Eugenia Budin, Ruxandra-Mioara Râjnoveanu, Doina Adina Todea

**Affiliations:** 1 Department of Pneumology, Iuliu Hațieganu University of Medicine and Pharmacy, 400332 Cluj-Napoca, Romania; vulturar.damianamaria@elearn.umfcluj.ro (D.-M.V.); dtodea@umfcluj.ro (D.A.T.); 2 Oncology and Palliative Care Department, George Emil Palade University of Medicine, Pharmacy, Science and Technology of Târgu Mureș, 540139 Târgu Mureș, Romania; corina.marginean@umfst.ro; 3 Pathophysiology Department, George Emil Palade University of Medicine, Pharmacy, Science and Technology of Târgu Mureș, 540142 Târgu Mureș, Romania; corina.budin@umfst.ro; 4 Palliative Medicine Department, Iuliu Hatieganu University of Medicine and Pharmacy, 400012 Cluj-Napoca, Romania; ruxandra.rajnoveanu@umfcluj.ro

**Keywords:** telemedicine, healthcare system, pandemic, armed conflict, SWOT analysis, digital health

## Abstract

**Background and Objectives:** Unlike previous studies that have examined telemedicine primarily in pandemic contexts, this research adopts a dual-crisis perspective, assessing perceptions during both pandemics and armed conflicts. Recent health crises, including the COVID-19 pandemic and armed conflicts, have exposed vulnerabilities in health systems and underscored the need for evidence-based strategies to enhance resilience. Telemedicine has emerged as an essential tool for ensuring continuity of care, mitigating workforce shortages, and improving access for vulnerable groups. This study examines healthcare professionals’ perceptions of telemedicine in Romania across two exceptional contexts—pandemics and armed conflict—focusing on applicability, systemic integration, and limitations. **Methods:** A cross-sectional descriptive survey was conducted among 409 healthcare professionals. Perceptions were analyzed using a SWOT framework and descriptive statistics (percentiles, median) to identify strengths, weaknesses, opportunities, and threats in both scenarios. **Results:** Perceptions of telemedicine were shaped by prior experience, with strong support for its use in both contexts. Strengths included adaptability and improved access, while weaknesses highlighted technical barriers, insufficient training, and the risk of clinical errors. Threats referred to poor coordination across facilities and cultural or language barriers. Opportunities highlighted the potential for digital infrastructure and integration into preparedness frameworks. **Conclusions:** Telemedicine is positioned as a strategic tool for strengthening national health resilience. Adaptive, context-sensitive policies, combined with investments in infrastructure and workforce capacity, are essential for integrating telemedicine into emergency preparedness and ensuring sustainable, inclusive responses to future crises. This dual-crisis approach represents the novelty of our study, demonstrating how telemedicine can serve as a strategic tool for resilience in both epidemiological and conflict-related emergencies.

## 1. Introduction

The COVID-19 pandemic and recent regional crises have emphasized the critical importance of resilient and adaptable healthcare systems worldwide. Among the solutions deployed, telemedicine has emerged as a key strategy for ensuring continuity of care under emergency conditions. It has demonstrated significant potential in bridging accessibility gaps, reducing exposure risks, and maintaining medical services when conventional delivery models are disrupted. The global health crisis profoundly transformed healthcare systems, accelerating the shift toward digital health and firmly establishing telemedicine as a core component of modern medical infrastructure. Despite the significant progress achieved, questions remain regarding the healthcare system’s current level of preparedness to deploy telemedicine effectively under new or evolving crisis conditions. While prior research has explored telemedicine from multiple angles, most studies have focused on civilian healthcare systems and single-crisis contexts. Recent international research has also addressed telemedicine in crisis settings. For instance, Vyas et al. (2022) [1] conducted a SWOT analysis on routine virtual outpatient departments, Tsegaye et al. (2023) [2] examined healthcare professionals’ views during the COVID-19 pandemic, and Harless et al. (2024) [3] investigated telehealth access barriers in underserved communities. In addition, Hu et al. (2025) [4] analyzed innovation networks in the advanced medical equipment industry to support regional digital health systems, while Li et al. (2025) [5] discussed digital applications in healthcare delivery. Although these studies provide valuable insights, they remain limited to single-crisis contexts and do not address dual scenarios or integrate both civilian and military perspectives. Australia’s telehealth support for Lebanon and Ukraine exemplifies feasibility in conflict zones but lacks a unified strategic framework [6]. In Romania, the rapid implementation of telemedicine during the pandemic revealed both innovation potential and persistent systemic weaknesses, such as technological disparities, regulatory limitations, and digital illiteracy. Between 2020 and 2021, the Romanian Ministry of Health implemented targeted measures to expand access to telemedicine services, particularly in urban and peri-urban areas [7]. Dascălu [8] further emphasized broader systemic weaknesses in Romania’s pandemic response, including deficits in infrastructure, coordination, and preparedness. However, a significant gap remains in the literature regarding the integrated analysis of telemedicine applicability in both pandemic and armed conflict scenarios, especially from the dual perspective of civilian and military healthcare professionals. National documents like the 2020 Activity Report of the Ministry of Health [7] (p. 1) and the SWOT analysis of digital services published by the Romanian Academy of Scientists [9] provide valuable insights; they remain limited in scope and do not address the comparative dimension or cross-sector perspectives essential for policy adaptation. Moreover, recent national academic contributions [10] explore health strategies and socio-economic consequences, yet they do not directly address healthcare professionals’ operational perceptions. International organizations have also emphasized the role of telemedicine in resilience frameworks—such as the WHO’s operational guidance for maintaining essential health services during COVID-19 [11] and the European Commission’s Digital Health and Care Strategy [12]—highlight the importance of digital resilience; they do not explicitly address telemedicine integration in dual-crisis contexts. This study used the SWOT framework (Gürel & Tat, 2017) [13]—originally developed as a strategic analysis tool to identify internal strengths and weaknesses, as well as external opportunities and threats, as a conceptual framework for organizing perception-based data. Unlike other studies that relied on this tool, this research introduced an adapted approach, based on descriptive statistics (percentiles), to systematically map quantitative survey results into strengths, weaknesses, opportunities, and threats, evaluating the implementation of telemedicine in both the pandemic and armed conflict contexts. The analysis of the degree of consensus was derived from the responses provided by healthcare professionals, both civilian and military, in Romania, which allows for a structured, data-based interpretation of how telemedicine is perceived in both contexts (pandemics/armed conflicts), with a focus on its applicability, limits, and potential for integration into the national healthcare system. This approach offers an intersectional, perception-based perspective that complements institutional strategies and contributes new insights to the literature on digital resilience in healthcare in exceptional circumstances, being closely correlated with the objectives of the study. The objectives of the study were as follows:

- To assess the degree of consensus among healthcare professionals, based on their academic and professional status and previous exposure to telemedicine, regarding the role of telemedicine, its applicability, and possible barriers to its integration into the national healthcare system.
- To assess the strengths, weaknesses, opportunities, and threats (SWOT) perceived by healthcare professionals in both contexts (pandemics/armed conflicts).
- To identify practical directions for integrating telemedicine into the national healthcare system and propose strategic recommendations to increase healthcare-system resilience.

## 2. Materials and Method

### 2.1. Study Design and Participants

**Figure 1:**
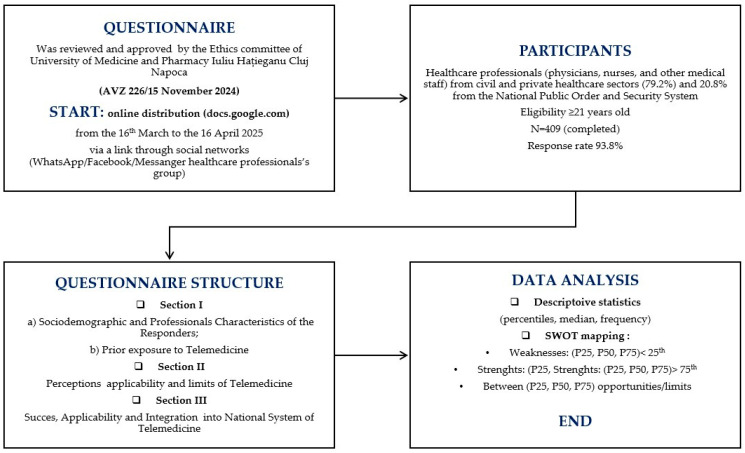
Flow chart of the study design.

This prospective observational study was designed and reported according to the STROBE checklist to ensure methodological transparency [14,15]. Data collection was carried out through a structured digital questionnaire entitled “Perceptions, Applications and Limitations in the Use of Telemedicine in Romania in Exceptional Situations (Pandemic and/or Armed Conflict)” (Supplementary Materials). Healthcare professionals (civilian and military) from Romania (physicians, dentists, medical assistants, communitarian assistant) were invited to participate. Access to the questionnaire was provided via a dedicated survey link shared on professional communication channels (email and closed social media groups). Participation was voluntary, anonymous, and GDPR-compliant, and it implied informed consent. Respondents were eligible if they were over 21 years of age and completed the survey. The reference population included approximately 169,200 healthcare professionals active in Romania in 2023 [16]. To determine the minimum required sample, standard formulas for large populations [17,18,19] were applied, with a 95% confidence level and 5% margin of error, resulting in a threshold of 384 participants. A total of 409 valid questionnaires were analyzed, exceeding the calculated requirement and ensuring representativeness, with a response rate of 93.9%. The overall design and participant flow are summarized in Figure 1.

### 2.2. Data Collection

Data were collected between 16 March and 16 April 2025 through a structured online questionnaire hosted on Google Forms. Distribution was carried out via institutional emails and professional social media groups (WhatsApp, Facebook, Messenger), ensuring voluntary and anonymous participation. All procedures were approved, together with the study protocol, by the Ethics Committee of the Iuliu Hațieganu University of Medicine and Pharmacy, Cluj-Napoca, Romania.

### 2.3. Ethical Considerations

The study was conducted in accordance with the Declaration of Helsinki and approved by the Ethics committee of the Iuliu Hațieganu University of Medicine and Pharmacy Cluj-Napoca, Romania (according to AVZ 226/15 November 2024). Participation was voluntary and anonymous, no identifying data were collected, and respondents were informed of their right to withdraw from the study at any time.

### 2.4. Measurements

The instrument was developed by the authors, and it consisted of three sections: (1a) sociodemographic and professional characteristics (age, gender, education, medical function, years of service), (1b) prior experience with telemedicine; (2) perceptions of telemedicine applicability, effectiveness, and limitations in pandemic and armed conflict contexts; and (3) views on the necessity of integration into the national health system and evaluation of its perceived success. Most items were assessed using a 5-point Likert scale (1 = strongly disagree to 5 = strongly agree), complemented by several open-ended questions that allowed respondents to provide qualitative insights.

### 2.5. Statistical Analysis

Descriptive statistics and visualizations, including boxplots, were generated using Microsoft Excel and JASP version 0.18.1 [20]. This study adopts a perception-based methodological approach, focusing on mapping trends and identifying consensus among respondents, rather than testing statistical significance. This choice reflects the exploratory nature of the research, focused on identifying patterns in healthcare professionals’ views of telemedicine during the COVID-19 pandemic and in conflict-related contexts. Likert-scale [21] responses were coded numerically (1–5) and summarized using percentile values (P25, P50, P75), which allowed non-parametric interpretation without assuming normality (Urdan, 2016) [22]. These thresholds were then systematically applied to construct the SWOT framework: items with scores above the 75th percentile were categorized as strengths, those below the 25th percentile were classed as weaknesses, and those between the 25th and 75th percentiles were interpreted as opportunities or threats depending on item content and context (pandemic vs. conflict). The SWOT model, traditionally used to assess internal strengths and weaknesses, alongside external opportunities and threats (Gürel & Tat, 2017) [13] (p. 2), was adapted here as a framework for organizing quantitative perception data. By integrating percentile-based summaries with strategic categories, the analysis allowed a structured interpretation of telemedicine’s applicability, barriers, and potential for integration into the Romanian healthcare system under exceptional circumstances.

This approach ensured that the SWOT analysis was directly data-driven, linking quantitative results with the strategic evaluation of telemedicine’s applicability, limitations, and potential integration into the national healthcare system.

## 3. Results

### 3.1. Participants’ Characteristics and Backgrounds

To appropriately assess the applicability and perceived limitations of telemedicine in exceptional situations—such as pandemics and armed conflicts—it is essential to contextualize the findings within the socio-demographic and professional characteristics of the respondents. Their background and prior exposure to telemedical services offer valuable insight into the relevance and consistency of their responses. The study included 409 validated responses, with a high completion rate of 93.88%. Respondents had a mean age of 41.02 years (SD = 9.2), with most falling within the 31–50 age group, indicating a predominantly mid-career professional profile. The average length of professional experience in healthcare was 11.89 years (SD = 7.79), and over 92% held advanced medical or academic titles. The majority were active in the civil and private healthcare sectors in Romania (79.2%), while 20.8% worked within the National Public Order and Security System (Table 1). Regarding prior exposure to telemedicine, a significant proportion of respondents reported having used or intended to use such services, particularly during or following the COVID-19 pandemic. A smaller percentage had experience with telemedicine in conflict areas or external missions (see Table 1). These characteristics reflect a highly experienced and professionally diverse group, offering a solid foundation for evaluating both the practical utility and the systemic challenges of telemedicine. This descriptive profile not only illustrates the credibility of the respondents’ perspectives but also serves as a valuable reference point for understanding how background variables such as professional experience, sector of employment, and academic training influence the perceptions analyzed in the following sections.

### 3.2. Healthcares’ Perceptions Based on Consensus Items Regarding Telemedicine Applicability, Potential for Integration into the National Healthcare System and Current National Framework

Based on the participant profile presented above, this subsection examines healthcare professionals’ perceptions of the effectiveness of telemedicine, the need for a national telemedicine program, and the role of regulatory frameworks. These dimensions provide insight into both the distribution of responses and the consistency of participants’ opinions, as presented in Table 2, where we can observe a strong consensus on the need for a national program to support the integration of telemedicine, with both percentiles positioned at the same point on the Likert scale (1) and an interquartile range of 1.0–5.0, with a standard deviation of ±1.01. In contrast, perceptions of the current regulatory framework, and the success of telemedicine reveal more variability. Respondents rated these aspects positively, although with a notable dispersion: the mean scores were 3.72 (±0.99 SD) for the regulatory framework and 2.71 (±1.03 SD) for the perceived success of telemedicine, on a Likert scale from 1 to 5, where 1 signifies strong agreement and 5 strong disagreement. The score of 2.71 reflects the widely recognized usefulness of telemedicine in both pandemic and conflict contexts, while the mean score of 3.72—closer to neutrality—indicates only partial agreement on the adequacy of the current regulatory framework, regardless of professional experience. Overall, these findings highlight broad professional support for the value of telemedicine in exceptional contexts, together with recognition of the need for more coherent and supportive regulation at the national level.

**Figure 2:**
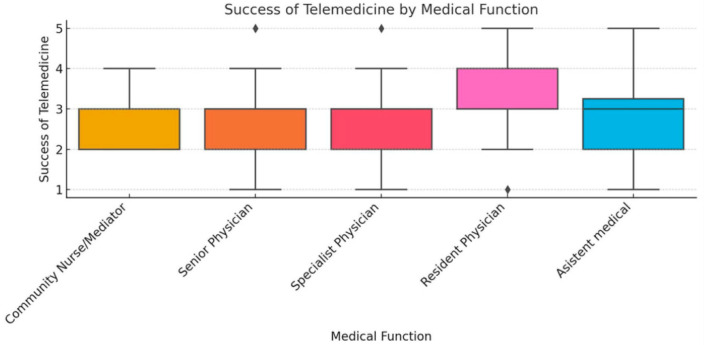
Success of telemedicine by medical function.

**Figure 3:**
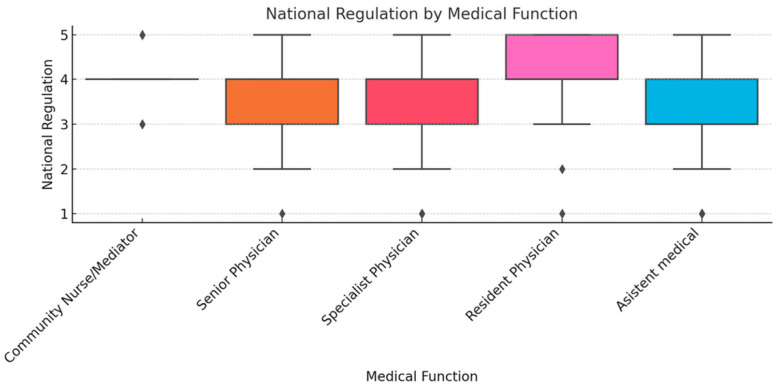
National regulation by medical function.

**Figure 4:**
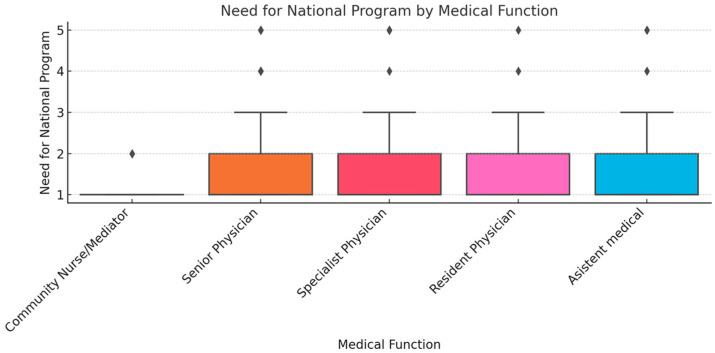
Need for national program by medical function.

**Figure 5:**
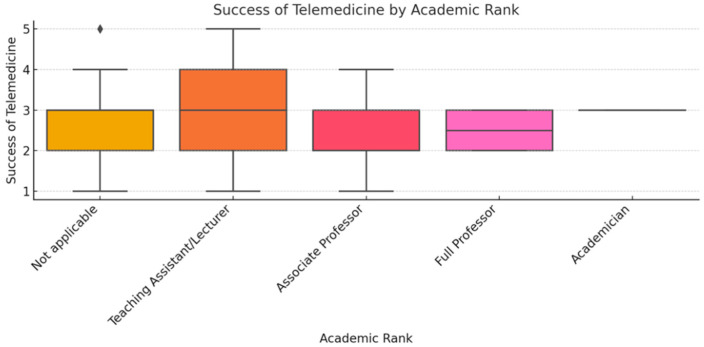
Success of telemedicine by academic rank.

**Figure 6:**
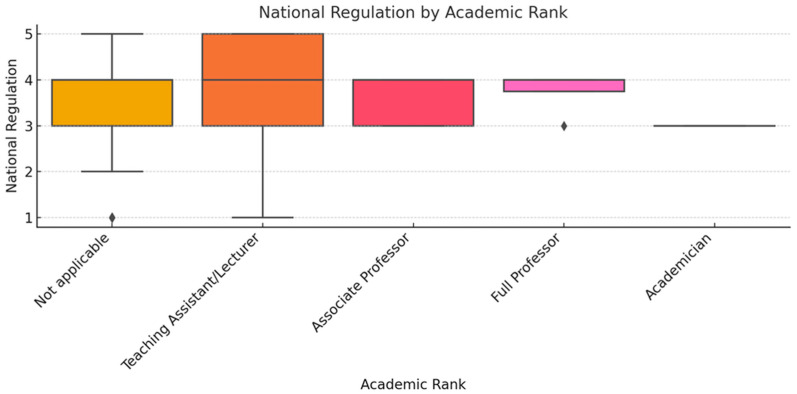
National regulation by academic rank.

**Figure 7:**
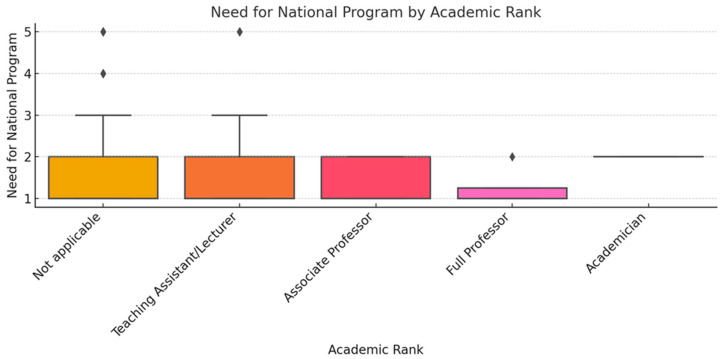
Need for national program by academic rank.

**Figure 8:**
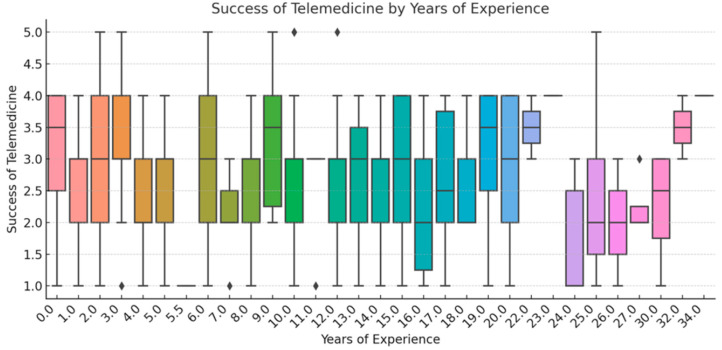
Success of Telemedicine by Years of Experience.

**Figure 9:**
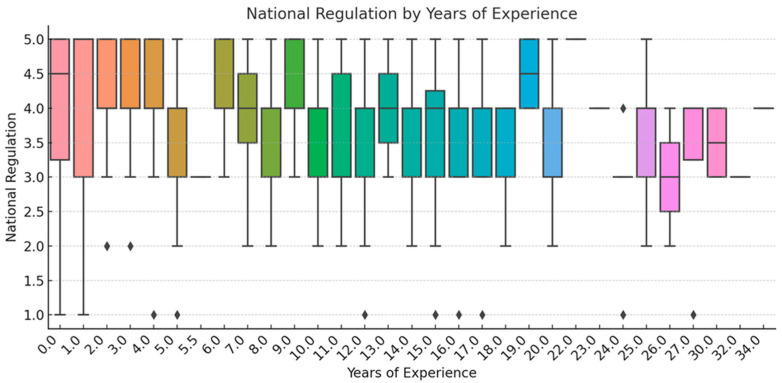
National regulation by years of experience.

**Figure 10:**
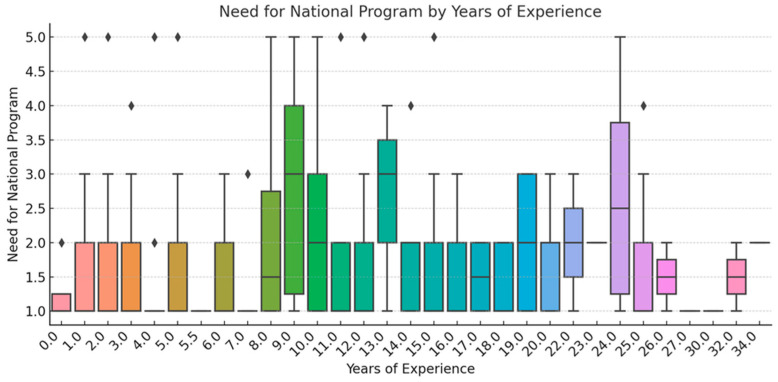
Need for national program by years of experience.

**Figure 11:**
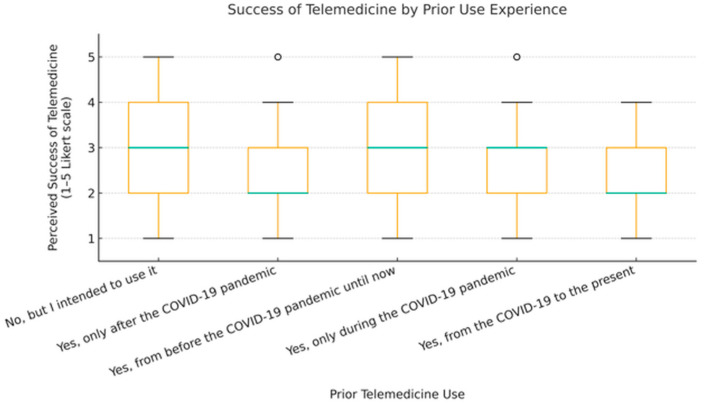
Success of telemedicine by prior telemedicine use.

**Figure 12:**
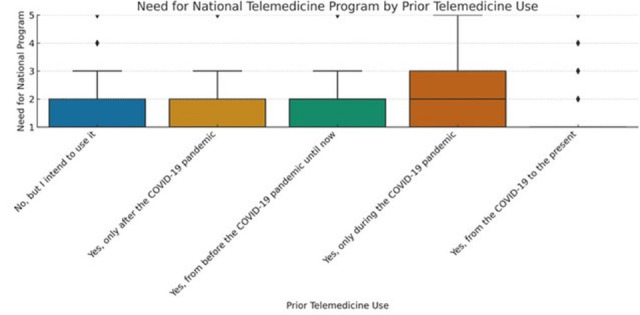
Need for national telemedicine program by prior telemedicine use.

To ensure analytical depth, responses were stratified by key characteristics, including years of experience, academic status, professional role, and previous exposure to telemedicine. This stratification was crucial for identifying patterns of agreement or disagreement in areas such as perceived success, regulatory clarity, and the need for institutional integration. Figure 2, Figure 3, Figure 4, Figure 5, Figure 6, Figure 7, Figure 8, Figure 9, Figure 10, Figure 11 and Figure 12 present distributions stratified by academic and professional status. Senior physicians report greater perceived success and more favorable evaluations of national regulations (Figure 2 and Figure 3), while the perceived need for a national program is consistently high across all roles (Figure 4). Academic professionals, particularly associate professors, display slightly more optimistic views of the success of telemedicine (Figure 5), while professors and lecturers perceive regulatory structures more positively (Figure 6). Across all academic ranks, support for a national program remains uniformly strong (Figure 7). Figure 8, Figure 9 and Figure 10 explore perceptions by years of experience. Professionals with more than 10 years of experience tend to report slightly higher median success scores and more favorable views of regulations (Figure 8 and Figure 9). However, the perceived need for a national program remains high across experience groups, with minor variability among respondents early in their careers (Figure 10). Figure 11 and Figure 12 examine the influence of prior use of telemedicine. Respondents with prior exposure consistently reported higher perceived success, stronger support for national integration, and greater trust in the regulatory framework. This trend suggests that direct experience with telemedicine fosters more favorable and consistent evaluations.

Figure 2, Figure 3, Figure 4, Figure 5, Figure 6, Figure 7, Figure 8, Figure 9, Figure 10, Figure 11 and Figure 12 provide visual representations of the distributions of responses across these variables, highlighting how perceptions differ across categories. From an academic perspective, senior physicians and academic professionals tended to express stronger agreement regarding the success of telemedicine and the need for a national program, while professionals early in their careers or those without prior experience in telemedicine presented more varied responses.

### 3.3. Healthcare Professionals’ Perceptions of Telemedicine in Exceptional Contexts Based on Descriptive Indicators and SWOT Classification

A dual context-based approach was used to capture how specific situational factors interact with professional training in shaping attitudes towards telemedicine. By analyzing percentiles (as an indicator of consensus), we were able to identify the benefits and limitations perceived by professionals regarding telemedicine in the two exceptional contexts, also providing an empirical basis for the strategic considerations discussed in the next section.

**Figure 13:**
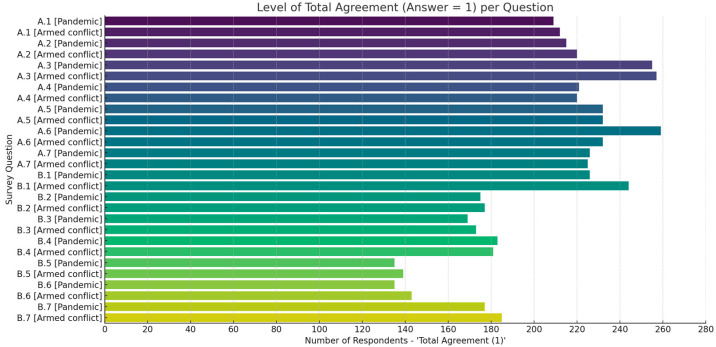
Level of consensus (total agreement) per question: synthesis from Table 3A,B.

To further assess perceptions of the benefits and constraints of Telemedicine in the two contexts, a descriptive analysis was performed using means, medians, and total number of agreements—classified by SWOT strengths (benefits) and weaknesses (limitations). Thus, in Table 3A, we present descriptive statistics for both contexts; at the level of items labeled “Strengths” and in Table 3B, we use descriptive statistics with items labeled “Opportunities”. We can observe a strong consensus, with over 200 (49.40%) respondents selecting strongly agree (Likert scale value, IQR 1 to 5, with [1—strong consensus] and [5—strong disagreement]) in both scenarios; see Figure 13 and Table 3A,B.

According to the data presented in Table 3A, we can notice that there was a strong consensus regarding the need to provide immediate medical communication (items A.3.1 [Pandemic]: 255 (62.34%) and A.3.2 [Conflict]): 257 (62.83%), to reduce the risk of infection (items A.6.1 [Pandemic]: 259 (63.32%) and A.6.2 (56.72%) to support the continuous monitoring of vulnerable patients (item A.2.1 [Pandemic]: 215 (52.56%) and A.2.2. [Conflict]): 220 (53.78%)). Other perceived benefits included cost and risk reduction (items A.5. in both contexts, 57%) and improved patient education (items A4 in both contexts, 54%). Regarding the constraint (limitations vs. opportunities) side (see Table 3B), the results highlighted a strong consensus regarding the perceived value of telemedicine in ensuring continuity of care and operational efficiency in resource-limited settings. Among the identified limitations and areas requiring improvement, as can be seen in Table 3B, were the items labeled “Opportunities”, such as coordination between field hospitals and emergency centers (item B.5.1 [Pandemic]: 135 (33%) and B.5.2. [Conflict]: 139 (34%) and almost the same procents of answers regarding the strong consensus with difficulty in providing care to vulnerable populations (item B.6.1: 33% and B.6.2: 34.9%). These items received less strong responses, reflecting lower consensus and indicating systemic gaps. In addition, moderate levels of agreement were observed for items related to technical barriers (item B.1), ethical concerns (item B.2), and the potential for medical errors (item B.3), training education (item A.4), and improved resource optimization (item A.7); see Table 3A,B. Figure 13 illustrates the overall level of agreement (value = 1) for all items of the questionnaire in the two contexts. The highest consensus was recorded for the items related to the applicability of telemedicine (items A.1–A.6), especially for urgent communication, infection control, and patient monitoring, for which over 230 respondents expressed full agreement. In contrast, the items addressing structural barriers and limitations (items B.1–B.7) showed lower but still constant levels of agreement, ranging between 160 and 185 responses, indicating recognition of challenges such as technical issues, ethical risks, and coordination gaps. These results confirm that healthcare professionals expressed strong alignment on the usefulness of telemedicine while also acknowledging significant constraints in terms of implementation. Differences between the two scenarios were modest but noticeable: for instance, infection control (A.6) and the continuous monitoring of vulnerable patients (A.2) achieved slightly higher agreement in pandemic settings, while barriers such as limited accessibility for vulnerable groups (B.4, B.6) were more frequently emphasized in conflict contexts (Table 3A,B). These results indicate a cautious recognition of the risks, rather than a complete rejection of telemedicine approaches.

In conclusion, the findings in Table 3A,B and Figure 13 confirm broad support for integrating telemedicine in exceptional contexts while underlining the importance of infrastructure investments, intersectoral coordination, and personalized support for vulnerable groups. These insights inform the strategic considerations detailed in the subsequent SWOT analysis (Table 4), insights that can contribute to national emergency preparedness and the development of digital health policies.

### 3.4. Synthesis of Telemedicine in Crisis Scenarios: SWOT Analysis

To consolidate the insights derived from both descriptive and evaluative data, a structured SWOT analysis was developed. This framework integrates item-level quantitative indicators presented in Table 3A,B. Strengths and applicability items (Table 3A) and perceived barriers and structural limits (Table 3B) were systematically mapped into SWOT categories using percentile thresholds (P25, P50, P75) and contextual interpretation. This ensured that the SWOT matrix reflected not only thematic interpretation but also quantitative evidence. The classification of Likert-scale items into the SWOT matrix was informed by percentile thresholds and contextual interpretation. Items with mean values below the 25th percentile or low total agreement scores were categorized as weaknesses, while those with high levels of consensus or means above the 75th percentile were designated as strengths. Intermediate or context-dependent items were classified as opportunities or threats, based on their policy implications and perceived impact on system resilience. The synthesized findings are summarized in Table 4, offering a strategic overview of the internal and external factors influencing telemedicine’s perceived effectiveness and long-term viability in exceptional healthcare scenarios.

These findings are particularly robust in the context of pandemics, where consistency in responses was highest (Table 3, Items A.2–A.6; see also Table 2, P25–P50 = 1.0 for national program support). In contrast, perceived weaknesses included concerns about the variability in telemedicine success (Table 2—P50 = 3.0), technical and infrastructure limitations (item B.1), and difficulties in providing care to vulnerable groups, particularly in conflict areas (items B.4 and B.6). Notably, professionals also reported inconsistencies in implementation and impact, as evidenced by the moderate dispersion of percentile values (Table 2—P25 = 2.0, P50 = 3.0, P75 = 3.0). Several opportunities were also identified, especially in areas requiring system-wide improvements, namely actionable areas for strengthening telemedicine infrastructure and institutional integration: coordination between emergency actors (item B.5), the need for specific staff training (item B.7), and legal/regulatory standardization (Table 2—regulation P50 = 4.0). Finally, the analysis highlighted several threats that may hinder sustainable implementation, such as ethical and data security concerns (Point B.2), risks of clinical error in the absence of physical examinations (Point B.3), and systemic challenges, such as regulatory ambiguity and fragmented inter-institutional coordination (Table 2—P50 = 4.0; Table 3—B.5).

This analytical approach identified several key strengths of telemedicine in both contexts, such as improved communication with healthcare professionals, improved infection control, the continuous monitoring of vulnerable patients, reduced costs and risks, and increased efficiency in patient education and resource management. The identified threats underline the importance of coherent governance and cross-sectoral alignment in future telemedicine policy efforts.

## 4. Discussion

This study highlights the strategic role of telemedicine as an evidence-based tool for strengthening national health resilience. The present analysis reveals significant correlations between previous exposure to telemedicine and favorable perceptions of its effectiveness, as well as the need for a structured national framework. Respondents with greater professional experience or holding high-ranking academic and institutional positions consistently reported more positive assessments. Moreover, a substantial proportion of respondents (85%) expressed strong agreement concerning the integration of telemedicine into the national health system. These findings highlight the urgent need to develop clear national regulations, implement specific training programs, and establish a coherent national strategy in the field of telemedicine. Integrating telemedicine into Romania’s national health strategy is not just a technological update but a decisive step towards a resilient, equitable, and future-proof health system. Such integration is essential not only to ensure continuity of care during pandemics and armed conflicts but also to develop long-term capacities to respond effectively to future public health emergencies. Building on this consensus, it is also important to distinguish how perceptions varied between pandemic and conflict contexts, as these differences provide additional insight into the contextual adaptability of telemedicine. These comparative results further indicate that, while the perceived strengths of telemedicine—such as infection control and patient monitoring—are consistently recognized, they were more highly valued in pandemic contexts, where infection prevention was a central concern. In contrast, barriers related to accessibility and equity were more frequently highlighted in conflict contexts, reflecting the resource constraints and coordination difficulties typical of these environments. Similar patterns have been reported in international studies, which have shown how contextual factors, such as infrastructure availability and institutional coordination, shape the perceived feasibility of telemedicine (Tsegaye et al., 2023; Harless et al., 2024) [2,3] (p. 2). Therefore, our findings reinforce the importance of adapting telemedicine strategies to the specific challenges of each type of crisis. This study also presents some limitations that should be considered when interpreting the results. First, the reliance on self-reported data through Likert-type items may introduce subjective bias, as individual responses could be influenced by institutional context, prior exposure to telemedicine, or personal attitudes toward innovation. Second, although the sample size is substantial, it may not fully capture the diversity of the healthcare system, particularly in underrepresented sectors or geographically isolated areas. This may limit the generalizability of findings, especially in settings affected by infrastructural or human resource disparities. Third, while Likert scales are suitable for capturing perceptions, they may not fully reflect the complexity of systemic challenges such as regulatory fragmentation or interoperability issues. The use of percentile values (P25, P50, P75) allowed for a more nuanced interpretation of the data; however, the observed variability in certain items suggests divergent viewpoints and a lack of consensus across the professional field. Finally, the study captures perceptions at a specific historical moment, shaped by recent health emergencies and security challenges. As telemedicine systems evolve—through policy updates, technological development, and wider adoption—future evaluations may reveal different patterns. Despite these limitations, the integration of the SWOT analysis using descriptive statistics to evaluate the implementation of telemedicine in both the pandemic and armed conflict contexts provides a robust overview of the strategic potential of telemedicine in emergency settings. The strong support for a national telemedicine program, coupled with concerns about regulation, coordination, and equitable access, underscores the need for coherent public health strategies. Furthermore, the widespread positive perception—even among professionals without extensive practical experience—indicates a high level of preparedness that can be supported through specific training, regulatory clarity, and digital inclusion policies.

Future research should extend the current findings through mixed-method approaches, integrating quantitative data with qualitative insights derived from interviews or focus groups. Particular attention should be paid to the experiences of marginalized and underserved communities, particularly those living in rural or conflict-affected areas, to better assess the role of telemedicine in alleviating health inequalities. In addition, investigations into the integration of emerging technologies—such as artificial intelligence and mobile health solutions—can provide innovative responses to persistent structural challenges. Evaluating long-term outcomes, including continuity of care, the effectiveness of training interventions, and overall cost-effectiveness, will be essential to inform sustainable national telemedicine policies.

## 5. Conclusions

The findings of this study underscore a broad consensus among Romanian healthcare professionals regarding the strategic relevance of telemedicine in exceptional contexts, particularly during pandemics and armed conflicts. Telemedicine is widely regarded as a critical tool for strengthening emergency preparedness, ensuring continuity of care, and mitigating infection risks. Previous experience with telemedicine appears to positively influence perceptions. Respondents who reported prior exposure to telemedicine during the pandemic expressed more consistent agreement across items, particularly for patient education (Item A.4) and infection control (Item A.6). In contrast, perceptions of the effectiveness of telemedicine in conflict scenarios showed slightly greater variability, reflecting the uncertainty of applying digital health solutions in unstable conditions. These findings suggest that familiarity with telemedicine increases confidence in its applicability, while a lack of prior experience may amplify perceived risks and limitations. High levels of agreement were recorded for its utility in facilitating urgent communication (Item A.3), monitoring vulnerable patients (A.2), and limiting disease transmission (A.6), highlighting its perceived effectiveness in high-pressure scenarios. A substantial majority of respondents—over 85%—endorsed the need for a dedicated national telemedicine program (Table 2, P25 = 1.0, P50 = 1.0), reflecting a strong alignment between practitioner perceptions and broader public health objectives. However, the moderate evaluation of telemedicine’s current success (Table 2, P50 = 3.0) and the variation in responses point to uneven implementation and gaps in system-wide integration. Several structural and operational barriers were identified, including insufficient training specific to emergency contexts, limited technological infrastructure in underserved areas, and a lack of standardized legal and procedural frameworks (Table 2—Regulation, P50 = 4.0). Cultural and linguistic challenges (B.4) and difficulties in delivering care to vulnerable populations (B.6) further constrain equitable access. Moreover, technical issues (B.1), ethical concerns (B.2), and diagnostic limitations in the absence of physical examination (B.3) were frequently acknowledged, underscoring the complexity of integrating telemedicine into routine practice. Ethical and regulatory challenges were among the most frequently cited barriers, particularly in relation to patient data confidentiality and liability in remote consultations. Respondents expressed concern about breaches of sensitive health information when telemedicine platforms are not fully secured or interoperable with institutional systems, and about the absence of clear legal responsibility in cases of diagnostic errors during online consultations. These findings highlight the need for robust regulatory frameworks and standardized protocols to ensure patient safety and professional accountability, while also addressing coordination gaps (B.5) and systemic fragmentation that hinder integration across healthcare facilities. These challenges reflect broader implementation gaps when compared to global standards, such as those outlined in the World Health Organization’s 2022 digital health report [11] (p. 2). While the strategic value of telemedicine is evident, particularly for synchronous interventions, its full potential remains underutilized in Romania due to regulatory fragmentation, limited interoperability, and a lack of institutional coordination. Our findings complement recent international evidence on the role of innovation in digital health. For example, Hu et al. (2025) [4] (p. 2) highlighted the contribution of innovation networks to strengthening regional digital health systems, while Li et al. (2025) [5] (p. 2) discussed the effectiveness of digital teaching strategies in pharmacology education, illustrating how digital tools can be applied in healthcare-related training and delivery. Although these studies do not address dual-crisis contexts, they reinforce the increasing relevance of digital transformation globally, while our study adds novelty by analyzing the perceptions of healthcare professionals in both pandemic and armed conflict scenarios.

Although these studies do not address dual-crisis contexts, they confirm the increasing relevance of digital transformation globally, while our study adds novelty by analyzing the perceptions of healthcare professionals in both pandemic and armed conflict scenarios.

In conclusion, the study highlights both the readiness and the constraints associated with the adoption of telemedicine. The strong support for national integration—despite practical limitations—emphasizes three strategic directions: (1) the need for a coherent regulatory and ethical framework tailored to crisis contexts, (2) investments in digital infrastructure and training for both civilian and military healthcare professionals, and (3) stronger interoperability and coordination across institutions. These recommendations build upon the WHO’s digital health frameworks while reflecting the specific challenges identified in Romania.

Together, these measures would enable telemedicine to evolve from a crisis-driven solution to a stable component of a resilient, equitable, and future-proof healthcare system.

## Figures and Tables

**Table 1:** Sociodemographic and professional characteristics of the responders.

Variable	No. (%)
**Age, mean ± SD**	41.02 (±9.2)
**Education**	
Post-university	38.8%
University	53.5%
Pre-university	7.6%
**Healthcare profession**	
Doctors	67.3%
Resident doctors	12.2%
Medical assistants	16.6%
Communitarian assistants	3.9%
**Medical system**	
National Private Sector	27.4%
National Civil Sector	51.8%
National Public Order and National Security System	20.8%
**Telemedicine experience**	
No, but I intended to	45.2%
Yes, during COVID-19 and until now	16.4%
Yes, starting with COVID-19 and until now	15.2
Yes, only during COVID-19 pandemic	13.2%
Yes, only after COVID-19 pandemic	10.0%
**Expertise in conflict area/TO missions**	
No	75.3%
Yes	24.7%
**Expertise with Telemedicine in conflict area/External missions—conflict area**	
No	90.5%
Yes	9.5%

**Table 2:** Descriptive statistics on key perceptions regarding the success of telemedicine, the actual framework, and the need to integrate telemedicine into the national program.

Items	Perceived National Regulation	Need for National Program	Perceived Success of Telemedicine
count	409	409	409
mean	3.72	1.7	2.71
P25	3.0	1.0	2.0
P50	4.0	1.0	3.0
P75	4.0	2.0	3.0
±S.D.	0.99	1.01	1.03

**Table 3:** Descriptive statistics of respondents’ perceptions regarding telemedicine in exceptional contexts (pandemic/armed conflict). (A) presents items classified as Strengths in the SWOT analysis, while (B) presents items classified as Opportunities.

Items	(A) Strengths and Applicability of Telemedicine in Exceptional Situations—Descriptive Indicators and SWOT Classification					
Question	Mean (Weighted Average)	Median (P50th)	Total Agreement		Percentile (P25th)	SWOT Category
			Number/% of Responders that Chose Value 1			
A.1.Do you consider that telemedicine helps cover the shortage of staff/specialists in hard-to-reach areas in the following situations?						
[Pandemic]	1.72	1.0	209	51.1%	1	Strength
[Armed conflict]	1.78	1.0	212	51.8%	1	Strength
A2. Do you consider telemedicine to enable the continuous monitoring of vulnerable patients, allowing early intervention in complications in the following situations?						
[Pandemic]	1.68	1.0	215	52.56%	1	Strength
[Armed conflict]	1.71	1.0	220	53.78%	1	Strength
A.3. Can telemedicine contribute to immediate communication with a doctor or consultant who can provide first aid instructions and/or assess the need for emergency care in the following situations?						
[Pandemic]	1.49	1.0	255	62.34%	1	Strength
[Armed conflict]	1.5	1.0	257	62.83%	1	Strength
A.4. Do you consider telemedicine to increase patient education and training in the following situations?						
[Pandemic]	1.67	1.0	221	54.03%	1	Strength
[Armed conflict]	1.7	1.0	220	53.78%	1	Strength
A.5. Do you consider telemedicine to reduce costs and risks (eliminating the need for vulnerable individuals to travel to dangerous areas) in the following situations?						
[Pandemic]	1.59	1.0	232	56.72%	1	Strength
[Armed conflict]	1.61	1.0	232	56.72%	1	Strength
A.6. Do you consider telemedicine to reduce the risk of spreading infectious diseases in the following situations?						
[Pandemic]	1.5	1.0	259	63.32%	1	Strength
[Armed conflict]	1.65	1.0	232	56.72%	1	Strength
A.7. Do you consider telemedicine to improve waiting times, document handling, and resource management (financial and personnel) in the following situations?						
[Pandemic]	1.62	1.0	226	55.25%	1	Strength
[Armed conflict]	1.66	1.0	225	55.01%	1	Strength
Question	Mean (weighted average)	Median (P50th)	Total Agreement		Percentile (P25th)	SWOT Category
	**(B) Perceived Barriers and Structural Limits to Telemedicine—Descriptive Indicators and SWOT** **Classification**					
**Question**	**Mean** **(weighted average)**	**Median** **(P50th)**	**Total Agreement**		**Percentile** **(P25th)**	**SWOT** **Category**
			**Number/% of responders that chose value 1**			
B.1. Do you believe that technical issues (*infrastructural gaps)* may be a barrier to the use of telemedicine in the following situations?						
[Pandemic]	1.6	1.0	226	55.25%	1	Strength
[Armed conflict]	1.55	1.0	244	59.65%	1	Strength
B.2. Do you believe that ethical risks *(lack of standardized frameworks: regulations, protocol standards, exposure of medical data to unauthorized people)* may be a barrier to the use of telemedicine in the following situations?						
[Pandemic]	1.87	2.0	175	42.78%	1	Strength
[Armed conflict]	1.89	2.0	177	43.27%	1	Strength
B.3. Do you believe that the use of telemedicine may increase medical errors *(*e.g., *inability to perform physical examination and insufficient training)* in the following situations?						
[Pandemic]	1.88	2.0	169	41.32%	1	Strength
[Armed conflict]	1.87	2.0	173	42.29%	1	Strength
B.4. Do you believe that a lack of education, as well as cultural and linguistic diversity (*cultural/linguistic barriers = digital literacy gaps*), may be a barrier to telemedicine use in the following situations?						
[Pandemic]	1.77	2.0	183	44.74%	1	Strength
[Armed conflict]	1.78	2.0	181	44.25%	1	Strength
B.5. Do you believe that coordination *(lack of protocols)* between field hospitals and emergency centers may be a barrier to telemedicine use in the following situations?						
[Pandemic]	2.15	2.0	135	33.00%	1	Opportunity
[Armed conflict]	2.12	2.0	139	33.98%	1	Opportunity
B.6. Do you find it difficult to provide medical care via telemedicine to vulnerable patients in the following situations?						
[Pandemic]	2.17	2.0	135	33.00%	1	Opportunity
[Armed conflict]	2.11	2.0	143	34.96%	1	Opportunity
B.7. Do you believe that the lack/insufficient training of medical staff for these situations (pandemic/armed conflict) may be a barrier to the use of telemedicine?						
[Pandemic]	1.82	2.0	177	43.27%	1	Strength
[Armed conflict]	1.8	2.0	185	45.23%	1	Strength
Question	Mean (weighted average)	Median (P50th)	Total Agreement		25th Percentile	SWOT Category

**Table 4:** SWOT: perceptions, insights, and statistical indicators.

Strengths	Opportunities
High consensus on emergency communication, infection control, and patient monitoring (Table 3: A.2–A.6) Strong support for national telemedicine implementation (Table 3: P25–P50 = 1.0) Infection control via telemedicine (A.6—259 [Pandemic], 232 [Conflict]) Continuous monitoring of vulnerable patients (A.2—215 [Pandemic], 220 [Conflict] Cost and risk reduction (A.5—232/232 total agreement) Patient education (A.4—mean ~1.68) Improved resource optimization (A.7—mean 1.62/1.66, agreement 226/225)	Improvement in coordination mechanisms (Item B.5) Investment in staff training (Item B.7) Legal and regulatory standardization (Table 3—regulation P50 = 4.0)
**Weaknesses**	**Threats**
Variability in the perceived success of telemedicine (Table 3—P50 = 3.0) Limited accessibility for vulnerable groups (Items B.6, B.4) Technical infrastructure concerns (Item B.1—mean ~1.6, agreement 226/244) Higher dispersion in success ratings (Table 3—P25 = 2.0, P50 = 3.0, P75 = 3.0)	Ethical and data security risks (B.2—P50 = 2.0, total agreement 175/177) Risk of errors without physical examinations (Item B.3—total agreement 169/173) Perceived regulatory ambiguity (Table 3—P50 = 4.0) Low institutional coordination (Item B.5—135/139) Policy fragmentation and institutional inertia (Table 3—P50 = 4.0; Table 3—B.5)

## Data Availability

The database used and analyzed during the current study is available from the corresponding author on reasonable request. Individual-level data cannot be made publicly accessible to protect participants’ anonymity and due to the authors’ intention to use the same database for further research.

## Supplementary Materials

The following supporting information can be downloaded at: https://www.mdpi.com/article/10.3390/healthcare13192474/s1, Survey: “Perceptions, Applications and Limitations in the Use of Telemedicine in Romania in Exceptional Situations (Pandemic and/or Armed Conflict)”.
