# Supplementary material for: Telemedicine in Times of Crisis—A SWOT Assessment Based on Romanian Healthcare Professionals’ Perceptions
Source: Healthcare (Basel). 2025 Sep 29;13(19):2474. doi: 10.3390/healthcare13192474 (PMC12523996; doi:10.3390/healthcare13192474)
Supplement: Supplementary file 1 [file healthcare-13-02474-s001.zip › healthcare-3854045-supplementary.pdf]

Survey: "Perceptions, Applications and Limitations in the Use of Telemedicine in Romania in Exceptional Situations (Pandemic and/or Armed Conflict)"

The undersigned, Ene Gianina, PhD student at UMF Iuliu Hatieganu Cluj Napoca, propose this study with the aim of identifying the general perception of healthcare professionals regarding the applicability and main limits in the use of telemedicine in the event of the declaration of an exceptional situation at national level (pandemic and/or armed conflict).
Your identification data (IP/email) are not processed or stored. Participation in the questionnaire is completely voluntary and anonymous, thus not subject to the GDPR. The decision to participate or not in this questionnaire will not interfere in any way with your rights as a healthcare professional. By completing the questionnaire you give your consent to participate in the proposed survey.

I ask you to support me in this endeavor!
Thank you!
Study period: March 16, 2025-April 16, 2025.

Section I. (a) Socio-demographic data and level of education - This section is dedicated to collecting information regarding the level of education

Q1. Age category *(Single answer)*
a) 20 - 29
b) 30 - 39
c) 40 - 49
d) 50 - 59
6) ≥ 60

Q2. The environment where you carry out your professional activity *(Single answer)*
a) urban
b) rural

Q3 **.** Education (last form of education completed) * *(Single answer)*
a) Vocational schools
b) High school
c) Faculty
d) Master's degree
e) Doctorate
f) Post-doctorate

Q4. Medical function *(Single answer)*
a) Medical assistant
b) Community health nurse/health mediator
c) Resident physician
d) Specialist doctor
e) Primary care physician

Q5. Academic degree *(Single answer)*
a) Assistant/University Lecturer
b) University lecturer
c) University professor
d) Academician
e) Others
e) Not applicable (N/A)

Q6. Health services provided *(Single answer)*
a) Community assistance services for the rural population and vulnerable population groups[1]
b) Preventive and control services[2]
c) Primary healthcare services
d) Specialized outpatient healthcare services
e) Hospital healthcare services
f) Rehabilitation, palliative and long-term care services
g) Emergency medical services

Q7. Length of service in the specialty (years of service): please <u>specify length of service (years)</u>

Q8. Professional affiliation (employed/former employee within the National System of Public Order and National Security) *(Single answer)*
yes
b) no

---

[1] Vulnerable groups: patients located in isolated rural areas without access to medical services, elderly patients unable to move, chronic patients with priority conditions (cardiopulmonary, metabolic, renal, etc.), terminally ill patients, patients with communicable diseases, rare diseases, cancer, patients with physical disabilities or special needs and patients with mental illnesses, including Roma citizens

[2] Preventive and control services to identify the risks of non-communicable diseases and to respond to the information and counseling needs of individuals, especially those at high risk or disadvantaged: counseling and education services for the health and nutrition of women and children, counseling and family planning/reproductive health, counseling for vaccination, counseling for the correction of harmful behaviors, services for transfusion security, preventive services for the surveillance of nosocomial infections; services for the prevention of mental illnesses

Q9. If you answered YES to the previous question, in which system? *(Single answer)*
a) Ministry of National Defence
b) Ministry of Interior
c) SRI
d) SPP
e) Ministry of Justice
f) other (please specify).....

Section I (b1) Expertise in the field
Objective: Telemedicine evaluation: knowledge, attitudes and preferences

Q1. Have you provided medical services using telemedicine? *(Single answer)*
a) never
b) yes, before the COVID-19 pandemic and until now
c) yes, only during the COVID-19 pandemic
d) yes, starting with the COVID-19 pandemic and until now
e) yes, only after the COVID-19 pandemic

Q2. If you answered YES to the previous question, for what purpose? *(Single answer)*
a) follow-up/monitoring-interpretation of laboratory results and data
b) in emergencies
c) in quarantine
d) other reasons, please specify....

Q3. What were/are the means of communication used? *(Multiple answers)*
a) synchronous (real-time video conferencing via digital applications: google meet, skype, zoom, etc.)
b) synchronous through dedicated platforms independent/integrated with the office's IT systems
c) synchronous via teleconsultations (telephone/video consultations)
d) asynchronous (sending a triage form/chat/website questionnaire, which is answered within 24 hours with the possibility of resolving it through a message, prescription, referral or appointment for a consultation at the office or for a synchronous consultation)
e) asynchronous for the transmission of imaging data with their storage and interpretation
f) asynchronous with remote biometric data transmission via bluetooth/telemonitoring (monitoring of vital functions, physical activity, etc.)

Q4. Please select the categories of patients for whom you most frequently provided consultations via telemedicine *(Multiple answers)*
a) stable chronic patients who required periodic monitoring

b) chronic patients with priority conditions
c) terminally ill patients
d) patients with infectious-contagious conditions/transmissible diseases
e) immunosuppressed patients (cancer/rare diseases/HIV/AIDS...)
f) pregnant women
g) children
h) patients with physical disabilities or special needs
i) patients with mental illnesses[3]
j) medical emergencies
k) other categories of patients: please specify

Q5. During telemedicine consultations, have you had to ask for support from more experienced colleagues ? *(Multiple answers)*

a) there was no need,
b) yes, on the website/platform
b) yes, on the phone
c) yes, via the internet
d) yes, other means (specify)

Q6. If you answered yes to the above question, how often have you been confronted with medical cases/situations outside your specialization? *(Single answer)*
a) daily
b) weekly
c) monthly
d) I don't know/I don't remember

Q7. On a scale of 1 to 5, where 1 means total disagreement and 5 total agreement, please quantify how satisfied you are with the way you communicate with patients using telemedicine services, in current practice.

Section I (b.2)- Expertise in the field

Objective: Assess knowledge, attitudes and preferences for armed conflict situations

Q1. Have you had the opportunity to actually participate in missions in operating theaters in the country or abroad or to participate in missions during the pandemic (mobile health

[3] mental illnesses: (anxiety disorders, mood disorders, personality disorders, somatization disorders, cognitive disorders (dementia)

centers/permanent/vaccination centers or, as the case may be, trauma departments or centers)? *(Single answer, as the case may be)*
yes
b) no

Q2. Have you had the opportunity to participate in training/instructions that had as a scenario the simulation of armed conflict and/or pandemic situations?
yes
b) no

Q3. Have you participated in missions specific to your training outside the national territory as a specialist under the auspices of international organizations (WHO, UNICEF, Red Cross, Doctors Without Borders, etc.) other than NATO, EU? *(Single answer)*
yes
b) no

Q4. If you answered YES to one of the previous questions, specify the number of missions/trainings performed:

Q5. During these missions, did you have the opportunity to practice telemedicine services? *(Single answer)*
yes
b) no

Q6. If you answered YES to the previous question, for what purpose did you carry out telemedicine activities *(Single answer)*
a) follow-up/monitoring (interpretation of results and laboratory data)
b) in emergencies
c) in quarantine
d) other reasons, please specify....

Q7. On a scale of 1 to 5, where 1 means total disagreement and 5 means total agreement, please quantify *how satisfied you were with the way you communicated with patients using telemedicine services,* in the situations described (missions in theaters of operations/missions during the pandemic/instruction/training missions).

Section II. Perceptions regarding the use of telemedicine in the event of the declaration of an exceptional situation (pandemic and/or armed conflict).

Objective 1: Identifying respondents' perceptions regarding the applicability of telemedicine for the situations described
Objective 2: Identifying respondents' perceptions regarding the main limits in the use of telemedicine for the situations described

II.1. Perceptions regarding the applicability of telemedicine at the national level, for the situations described

Q1. On a scale from 1 to 5, where 1 means total disagreement and 5 means total agreement, please quantify to what extent you consider that *telemedicine contributes to covering the shortage of personnel/specialists, in hard-to-reach areas,* in the following situations **:**
a) pandemic *(Scale 1-Scale 5)*
b) armed conflict *(Scale 1-Scale 5)*

Q2. On a scale of 1 to 5, where 1 means total disagreement and 5 means total agreement, please quantify to what extent you consider that *telemedicine contributes to the continuous monitoring of vulnerable patients* , allowing early intervention in case of complications, in the following situations:
a) pandemic *(Scale 1-Scale 5)*
b) armed conflict *(Scale 1-Scale 5)*

Q3 . On a scale from 1 to 5, where 1 means total disagreement and 5 means total agreement, please quantify to what extent you appreciate that telemedicine *contributes to immediate communication with a doctor or a medical consultant who can provide first aid instructions and/or determine the need for emergency assistance (or not)* in the following situations:
a) pandemic *(Scale 1-Scale 5)*
b) armed conflict *(Scale 1-Scale 5)*

Q4. On a scale of 1 to 5, where 1 means total disagreement and 5 means total agreement, please quantify to what extent you consider that *telemedicine contributes to increasing the level of education and training of patients* in the following situations *:*
a) pandemic *(Scale 1-Scale 5)*
b) armed conflict *(Scale 1-Scale 5)*

Q5. On a scale of 1 to 5, where 1 means total disagreement and 5 means total agreement, please quantify to what extent you consider that *Telemedicine helps reduce costs and associated risks* (eliminating the need to move vulnerable people to dangerous areas) in the following situations *:*
a) pandemic *(Scale 1-Scale 5)*
b) armed conflict *(Scale 1- Scale 5)*

Q6. On a scale from 1 to 5, where 1 means total disagreement and 5 means total agreement, please quantify to what extent you consider that *telemedicine contributes to reducing the risk of transmission of infectious diseases* in the following situations *:*
a) pandemic *(Scale 1- Scale 5)*
b) armed conflict *(Scale 1-Scale 5)*

Q7. On a scale from 1 to 5, where 1 means total disagreement and 5 means total agreement, please quantify to what extent you consider that telemedicine *contributes to better management of waiting times, documents and resources (financial and personnel)* in the following situations *:*
a) pandemic *(Scale 1-Scale 5)*
b) armed conflict *(Scale 1-Scale 5)*

II. 2. Limits on access to telemedicine in the situations described

Q1. On a scale of 1 to 5, where 1 means total disagreement and 5 means total agreement, please quantify to what extent you consider that the *technical issues,*[4] *may constitute an impediment to the use telemedicine,* in the following situations *:*
a) pandemic *(Scale 1-Scale 5)*
b) armed conflict *(Scale 1-Scale 5)*

Q2. On a scale from 1 to 5, where 1 means total disagreement and 5 means total agreement, please quantify to what extent you consider that *the associated ethical risks (regulations, protocol standards, possibility of exposing medical information to unauthorized persons) may constitute an impediment to the use of telemedicine,* in the following situations:
a) pandemic *(Scale 1-Scale 5)*
b) armed conflict *(Scale 1-Scale 5)*

Q3. On a scale from 1 to 5, where 1 means total disagreement and 5 means total agreement, please quantify to what extent you believe that *the use of telemedicine can lead to an increase in medical errors (impossibility of performing a physical examination)* in the following situations : :
a) pandemic *(Scale 1-Scale 5)*
b) armed conflict *(Scale 1-Scale 5)*

Q4. On a scale from 1 to 5, where 1 means total disagreement and 5 means total agreement, please quantify to what extent you consider that *lack of education, cultural and linguistic diversity can constitute an impediment in the use of telemedicine,* in the following situations:
a) pandemic *(Scale 1-Scale 5)*

[4] Technical issues - connectivity issues, technical equipment of mobile offices/centers as well as patients' ability to use technology, especially patients with special needs)

b) armed conflict *(Scale 1-Scale 5)*

Q5. On a scale from 1 to 5, where 1 means total disagreement and 5 means total agreement, please quantify to what extent you consider that *the need for coordination between field hospitals and emergency reception centers may constitute an impediment to the use of telemedicine* in the following situations:
a) pandemic *(Scale 1-Scale 5)*
b) armed conflict *(Scale 1-Scale 5)*

Q6. On a scale of 1 to 5, where 1 means total disagreement and 5 means total agreement, please quantify to what extent you consider it *difficult to provide of medical care through telemedicine services to vulnerable patients,* in the following situations:
a) pandemic *(Scale 1-Scale 5)*
b) armed conflict *(Scale 1-Scale 5)*

Q7. On a scale from 1 to 5, where 1 means total disagreement and 5 means total agreement, please quantify to what extent you consider that *the lack/insufficient training of medical personnel for these situations/environments (pandemic/armed conflict) may constitute an impediment to the use of telemedicine* .
a) pandemic *(Scale 1-Scale 5)*
b) armed conflict *(Scale 1-Scale 5)*

Section III – Perceptions regarding possible measures to optimize telemedicine at the national level

Q1. On a scale of 1 to 5, where 1 means total disagreement and 5 means total agreement , please quantify to what extent you consider *telemedicine to have been used successfully during the COVID-19 period?*

Q2. On a scale from 1 to 5, where 1 means total disagreement and 5 means total agreement, please quantify to what extent you consider that *telemedicine is optimally regulated at the national level.*

Q3. On a scale of 1 to 5, where 1 means total disagreement and 5 means total agreement, please quantify to what extent you consider *it necessary to create a specific national telemedicine program.*
